# Mutation detection and prenatal diagnosis of XLHED pedigree

**DOI:** 10.7717/peerj.3691

**Published:** 2017-08-28

**Authors:** Yao Lin, Wei Yin, Zhuan Bian

**Affiliations:** 1 The State Key Laboratory Breeding Base of Basic Science of Stomatology (Hubei-MOST) & Key Laboratory of Oral Biomedicine Ministry of Education, Wuhan University, Wuhan, Hubei, China; 2 Department of Endodontics, Xiamen Stomatology Hospital, Xiamen, Fujian, China

**Keywords:** Prenatal diagnosis, *EDA* gene, Methylation analysis, X-linked hypohidrotic ectodermal dysplasia, Mutation

## Abstract

**Background:**

The phenotypic characters of X -linked Hypohidrotic Ectodermal Dysplasia (XLHED) are the dysplasia of epithelial- and mesenchymal-derived organs. Ectodysplasin (*EDA)* is the causative gene of XLHED.

**Methods:**

The current study reported a large Chinese XLHED pedigree. The genomic DNA of adult and fetus was extracted from peripheral blood and shed chorion cell respectively. The nucleotide variation in *EDA* gene was screened through direct sequencing the coding sequence. The methylation state of *EDA* gene’s promoter was evaluated by pyrosequencing.

**Results:**

This Chinese XLHED family had two male patients and three carriers. All of them were with a novel *EDA* frameshift mutation. The mutation, c.172-173insGG, which leads to an immediate premature stop codon in exon one caused severe structural changes of EDA. Prenatal diagnosis suggested that the fetus was a female carrier. The follow-up observation of this child indicated that she had mild hypodontia of deciduous teeth at age six. The methylation level of *EDA* gene’s promoter was not related to carriers’ phenotype changes in this family.

**Discussion:**

We reported a new frameshift mutation of *EDA* gene in a Chinese family. Prenatal diagnosis can help to predict the disease status of the fetus.

## Introduction

Dysplasia of epithelial- and mesenchymal-derived organs which include tooth, sweat gland, hair and nail is called ectodermal dysplasias (EDs) (Itin & Fistarol, 2004). It can be inherited as autosomal dominant, autosomal recessive and X-linked recessive modes (X-linked recessive type, X-linked hypohidrotic ectodermal dysplasias, XLHED) (Priolo & Lagana, 2001). The prevalence of XLHED is about less than one in per 100, 000 (Chassaing et al., 2006).

Previous studies have confirmed that XLHED is caused by the nucleotide variation of Ectodysplasin A (*EDA)* gene. The *EDA* gene has at least nine transcripts because it has extensive alternative splicing (Kere et al., 1996). The longest isoform is EDA-A1 which encodes 391-amino acid (Döffinger et al., 2001; Headon et al., 2001). The variations in *EDA* gene are also detected in isolated hypodontia patients (Yin & Bian, 2015; Yin & Bian, 2016). There are more than 100 *EDA* gene mutations which have been detected in XLHED patients (Tao et al., 2006).

EDs can also present as part of a syndrome, which includes ectodermal dysplasia, hypohidrotic, with immune deficiency, ectodermal dysplasia and neurosensory deafness and ectodermal dysplasia with adrenal cyst. The causative gene for syndromic EDs includes *NEMO* and *TP63* gene (Yin et al., 2010).

In the current study, we reported a large Chinese XLHED pedigree. Nucleotide sequence analysis confirmed a new XLHED-related *EDA* frameshift mutation. Pyrosequencing analysis was used to study the *EDA* gene promoter’s methylation state of pedigree’s carriers. Furthermore, we performed the prenatal diagnosis for one pregnancy carrier.

## Materials & Methods

### Ethical approval

The protocol of this study was approved by the Institutional Review Board (IRB) of Hospital and School of Stomatology, Wuhan University (200702). All contents in this study were carried out following the approved guidelines. All participants or their guardians signed the written informed consents. This study followed the recommendation of STROBE guidelines (https://www.strobe-statement.org/index.php?id=strobe-home).

### Pedigree diagnosis and DNA sample collection

The proband was the patient of School and Hospital of Stomatology, Wuhan University. Physical examination was performed as thoroughly as possible by two dentists respectively. The panoramic radiograph film was used to evaluate the development of tooth and alveolar bone. Then other family members were interviewed and examined. The peripheral blood samples (10 ml) were collected using EDTA 2Na as anticoagulant. DNA was extracted from leukocytes using standard proteinase-K phenol chloroform methods (Yin et al., 2010).

### Polymerase chain reaction, mutation screening and protein structural analysis

The entire coding region and exon-intron boundaries of *EDA* gene were amplified and purified by the same way with our previous study (Yin, Ye & Bian, 2012). In brief, the coding exons and intron-exon boundaries of *EDA* gene were amplified with specific primers. The polymerase chain-reaction (PCR) purification kit (Omega, Norcross, GA, USA) was used to purify the amplified fragments.

The nucleotide sequence was detected with an ABI PRISM 3730 genetic analyzer and analyzed in BLAST database of the National Center for Biotechnology Information (NCBI). After identifying the nucleotide variant in *EDA* gene, two hundred unrelated healthy controls were recruited to exclude the possibility of Single nucleotide polymorphism (SNP). The protein structure of wild-type and mutant EDA protein were constructed by Swiss-pdbViewer 4.0 software.

### Prenatal diagnosis

Chorion cells were obtained with the help of ultrasonic at gestational age of 13 weeks. The QIAamp^®^ DNA Mini Kit (QIAGEN, Hilden, Germany) was used for DNA isolation from chorion cells. The determination of fetal’s gender was based on the detection of *SRY* gene. Primers for *SRY* genes are F: 5′-CATGAACGCATTCATCGTGTGGTC-3′ and R: 5′-CTGCGGGAAGCAACTGCAATTCTT-3′ (Shahid et al., 2004). The mutation detection was the same with “Polymerase chain reaction, mutation screening and protein structural analysis”.

### Methylation analysis of *EDA* gene promoter

The methylation state of *EDA* gene promoter was determined in the same way with our previous work (Yin et al., 2013). In brief, the CpGenome DNA Modification Kit (Intergen Company, Purchase, NY, USA) was used to perform the bisulfite modification of the genome. The sequence (“CGgctgaggcagaCGcagCGgctccCG”) in *EDA* gene promoter was used to determine the methylation state. After amplification, the product was sequenced with PYRO MARK ID (BIOTAGE).

### Statistical analysis

Chi-Square Test and ANOVA were used to analyze the differences in methylation state of *EDA* gene promoter. It was considered statistically significant when the *P* value < 0.05.

## Results

### Clinical features

**Figure 1:**
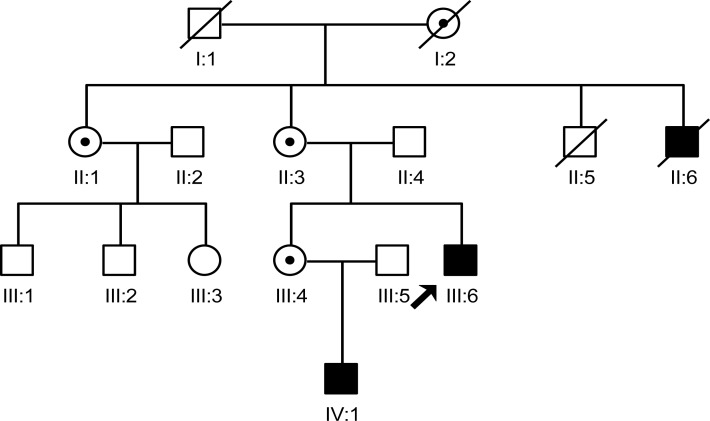
The pedigree gram of this Chinese XLHED family. The proband was indicated by the arrow. Symbols marked by a slash indicated that the subject was deceased. Males were indicated by squares, females were indicated by circles. Blackened symbols represented individuals who were identified as patients according to clinical examination. Carriers were indicated by a black dot. Family members (II:2, III:1, III:2 and III:3) were not involved in this study as they did not agree to publish any of their information.

In this study, we interviewed seven family members (II:1, II:3, II:4, III:4, III:5, III:6 and IV:1), in which two males were patients and three female were carriers. The two male patients share similar phenotype which includes the characteristic face of XLHED, hypohidrotic, and tooth agenesis. Three carriers only had abnormal shape of teeth and sparse hair (Fig. 1).

### Analysis of *EDA* gene mutation and protein structural changes

**Figure 2:**
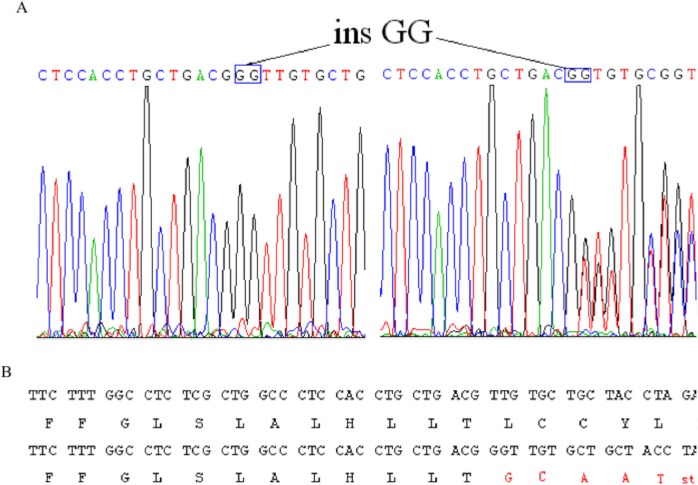
Identification of a novel 2-bp insertion mutation in the EDA gene. (A) DNA sequence of *EDA* gene exon 1 of the proband and an female carrier. Overlapping peak was caused by c.172–173insGG mutation; (B) Comparison wildtype DNA and protein sequences with mutant* EDA*. Mutant transcript stops at 62nd amino acid.

**Figure 3:**
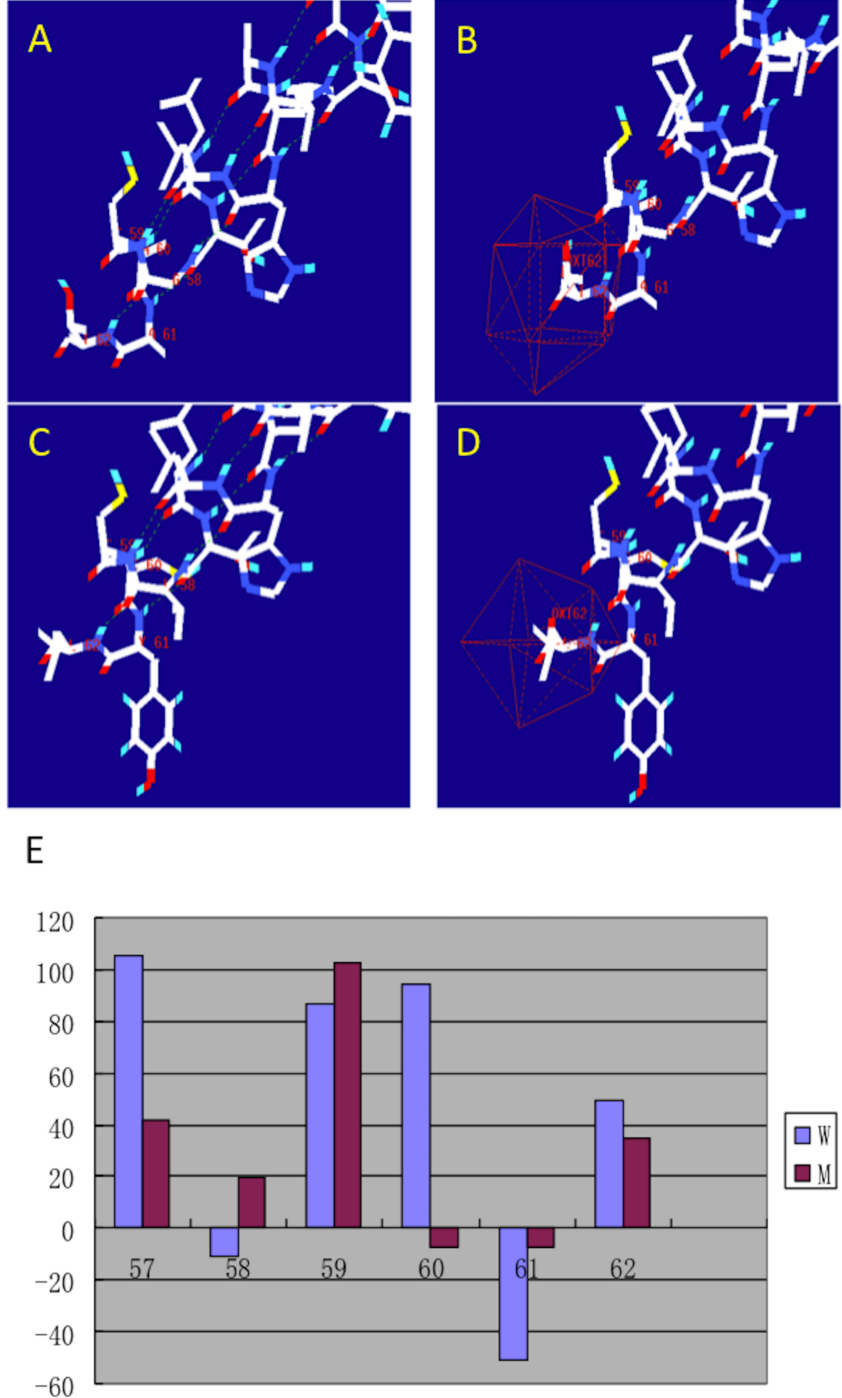
Structural analysis of the mutant and wild type EDA. The figure was generated as described in the text. (A) H-band distribution of mutant EDA; (B) Surface electrostatic potential of mutant EDA; (C) H-band distribution of the wildtype EDA; (D) Surface electrostatic potential of wildtype EDA; (E) Force field energy comparison of mutant (M) and wildtype (W) EDA. Red indicates mutant EDA, while blue refers to the wildtype.

After making the diagnosis, we aimed to determine the causative gene of this pedigree. We screened the coding sequence of the *EDA* gene. A new frameshift mutation, 171–172insGG, in exon one was identified in all patients and carriers (Fig. 2). All healthy members in this family do not have the frameshift mutation. The healthy controls did not have this variation in *EDA* gene. This frameshift mutation was predicted to result in an immediate premature stop codon in exon one. Mutant EDA only had 62 amino acids. According to the analysis of Swiss-pdbViewer software, the mutant residue located on the outer surface of the EDA protein. The structure of mutant EDA was more compact compared with that of wildtype EDA. The direction of H-band and electrostatic potential also greatly changed (Fig. 3).

### Methylation state of *EDA* gene promoter

In order to analyze the role of inactivation of X chromosome in changes of carrier’s phenotype, we performed methylation state analysis of *EDA* gene promoter for one carrier (III:4). The methylation frequency of four sites in *EDA* gene promoter was showed in Table 1. Compared to the 95% CI for methylation of each site (Yin et al., 2013), two out of these four sites of carrier III:4 exhibited hypermethylation state. While on site was in hypomethylation level.

**Table 1:** The methylation state of *EDA* gene’s promoter of family’s carrier (III:4).

Site	I	II	III	IV
95% CI	21.46–26.79	22.01–25.57	19.47–23.25	27.12–31.79
III:4	26.42	39.08	30.38	17.79

### Prenatal diagnosis

One of pedigree’s carriers (III:4) was pregnant after we determined the causative gene mutation of this family. With the informed consent, we performed prenatal diagnosis for her. Chorion cells were isolated under ultrasonic at gestational age of 13 weeks. The genomic DNA was immediately extracted. The short tandem repeat (STR) analysis was used to exclude the mixture of mother tissue in chorion cells. We determined that the fetus was female. The result was reliable which was confirmed by the positive and negative control. The nucleotide sequencing analysis indicated that the fetus was a carrier. We checked the health condition of this child every year. She had mild hypodontia of deciduous teeth at age six.

## Discussion

It is well known that XLHED patients have well-characterized phenotype. Patients suffer from the deformity of tooth, hair, skin, alveolar bone and suboriferous. The patients in this pedigree with frameshift mutation of *EDA* gene had similar characters with our previous XLHED patients who had missense mutation and nonsense mutation in different exons. Therefore we had the same conclusion with the previous report that there was no evident correlation between genotype and phenotype in XLHED patients (Yin, Ye & Bian, 2012).

EDA is a trimeric type II membrane protein and has intracellular domain, transmembrane domain, furin subdomain, collagen subdomain and tumor necrosis factor (TNF) homology subdomain (Schneider et al., 2001). The TNF homology domain plays a critical role in the homotrimerization of ligands and the binding to the receptor (Döffinger et al., 2001). The frameshift mutation, 171-172insGG, stopped EDA transcript at the 62nd amino acid. Interceptive EDA only had intracellular domain and transmembrane domain. Therefore, its ligand, EDAR, neither bind to it nor get the intracellular signals. Furthermore, mutant EDA was unable to form the trimer.

In XLHED pedigree, heterozygous carriers show only minor to moderate degrees of typical features (Cambiaghi et al., 2001). Aberrant methylation of *EDA* gene was thought to be a reasonable explanation because severe manifestations of female in X-linked recessive disorder might be related to skewing of X-inactivation (Lyon, 2002). Our previous study suggested that Chinese XLHED carriers were inclined to have a hypermethylated *EDA* promoter (Yin et al., 2013). Carriers with hypermethylated EDA promoter had a higher chance to suffer conical shaped tooth and nail dysplasia than carriers with hypomethylated EDA promoter. In the present study, female carriers also exhibited abnormal shaped teeth. Therefore, we also analyzed the *EDA* gene promoter’s methylation state of one female carrier (III:4). Our results suggested that two detected sites in *EDA* gene promoter were hypermethylated compared to that of healthy control. However, one of these four detected sites was in the hypomethylated level. Therefore, we speculated that there was other mechanisms involving in the regulation of XLHED carrier’s phenotype.

During the past several years, prenatal determination was practicable through DNA testing on embryo (Rigolet et al., 2007). While only few publications reported the prenatal diagnosis in XLHED pedigree. More studies were needed to summarize its merits. Our experience indicated that prenatal diagnosis which relied on chorion cells performed very well. The diagnosis can be applied very early, in the first trimester, during pregnancy. Therefore, it is a prerequisite for the optimal care of the carrier’s pregnant and her fetus.

## Conclusions

A novel *EDA* frameshift mutation was confirmed in a large Chinese XLHED pedigree. Mutant EDA only had 62 amino acids, which caused great changes compared with the wild-type EDA.

## Additional Information and Declarations

### Data Availability

The following information was supplied regarding data availability: The raw data is included in the Materials and Methods section and figures in the manuscript.
